# Measurement-Based Analysis of a Novel Method for Orthodontic Mandibular Molar Protraction Using an Improved Superelastic Ni-Ti Alloy Wire

**DOI:** 10.7759/cureus.89450

**Published:** 2025-08-05

**Authors:** Mayuka Watanabe, Ikuo Yonemitsu, Koji Saisho, Yuka Tanaka-Takemura, Naoaki Mikami, Mika Kikuchi, Takemura Hiroshi, Kohei Soga, Kazuhiro Suga, Motohiro Uo, Takashi Ono

**Affiliations:** 1 Department of Orthodontic Science, Graduate School of Medical and Dental Sciences, Institute of Science Tokyo, Tokyo, JPN; 2 Department of Mechanical and Aerospace Engineering, Faculty of Science and Technology, Tokyo University of Science, Tokyo, JPN; 3 Department of Medical and Robotic Engineering Design, Faculty of Advanced Engineering, Tokyo University of Science, Tokyo, JPN; 4 Department of Mechanical Engineering, Faculty of Engineering, Kogakuin University, Tokyo, JPN; 5 Department of Advanced Biomaterials, Graduate School of Medical and Dental Sciences, Institute of Science Tokyo, Tokyo, JPN

**Keywords:** 6-axis sensor, counter moments, improved superelastic ni-ti alloy wires, long hook, mandibular molar protraction, ni-ti closed coil spring, orthodontic measurement system, orthodontics, short hook, simulated teeth

## Abstract

Purpose

Improved superelastic nickel-titanium (Ni-Ti) alloy wires (ISWs) can be used not only for aligning but also for closing the extraction space. The objective of this study was to measure and compare the force and moment generated during mandibular molar protraction using an ISW combined with either short or long hooks under simulated crowded dentition conditions.

Materials and methods

Assuming crowded dentition following mandibular first premolar extraction, we designed a three-tooth model simulating the canine, the second premolar, and the first molar. First, 0.018 × 0.025-inch slot self-ligating brackets were bonded to the simulated canine and first premolar, and a tube was bonded to the first molar. Next, a 0.016 × 0.022-inch ISW was ligatured into the brackets. Either a long hook or a short hook was attached next to the first premolar bracket, and the hook and molar tube were pulled with a 150-gf Ni-Ti closed coil spring. We investigated the force and moments exerted on the teeth using an orthodontic simulator attached to a high-precision six-axis sensor.

Results and discussion

Using a short hook for molar protraction caused the simulated teeth to collapse into the extraction space, generating an insufficient counter moment (average moment: 2.0 ± 0.4 N·mm). In contrast, combining ISW with a long hook generated significantly (p<0.01) higher counter moments on the first molar (average: 6.5 ± 0.6 N·mm), resulting in controlled bodily movement without the need for rigid wires. While Ni-Ti wires are generally limited to leveling, our system utilizes a single archwire throughout all treatment stages. This approach demonstrated that early stage space closure is achievable by applying appropriate counter moments through molar protraction using the long hook.

## Introduction

In orthodontic treatment, the nickel-titanium alloy (Ni-Ti) archwire is widely used in leveling the crowded dentition at the initial stage due to its properties of shape memory and superelasticity [1,2]. These mechanical characteristics, including its constant load-deflection behavior and temperature-dependent phase transformation, have been extensively investigated [3]. Unlike stainless steel or beta-titanium wires, Ni-Ti archwires show a plateau in load-deflection curves, delivering relatively constant force over a wide deflection range, beneficial during initial orthodontic treatment [4]. On the other hand, the Ni-Ti archwire is not suitable for space closure due to low rigidity. Protraction of adjacent teeth causes the wire to deflect, resulting in tipping of the teeth toward the extraction space [5]. If the canine tips distally toward the extraction space, the wire will deflect, causing the anterior teeth to tilt as well, which will lead to bite deepening [6]. Therefore, the extraction-space closure would be commonly initiated by changing the archwire from the Ni-Ti archwire to a more rigid one after finishing the leveling of the teeth. In other words, space closure should not be started immediately after the premolar extraction at the beginning of treatment, rather it should be postponed until the leveling is finished [7].

In this treatment period, three-dimensional shrinking of the alveolar bone at the extraction site may progress, leading to limited tooth movement or extended overall treatment duration followed by slower tooth movement [8]. An improved superelastic Ni-Ti alloy wire (ISW) (L&H Titan, Tomy International Inc., Tokyo, Japan) has been used to align teeth and to close the extraction space simultaneously to avoid the unfavorable phenomenon in the alveolar bone. The ISW can be bent to produce adjustments including a V-shaped bend and a tip-back bend [9] with heat treatment [10] using a machine called SOARER X (Tomy International Inc.). The low rigidity of ISWs, when combined with a V-shaped bend, helps achieve a balance between the contraction force and the bend magnitude. This balance enables bodily tooth movement rather than tipping during space closure, especially when adjacent teeth are retracted using an elastomeric chain [11]. However, despite this compensatory mechanism, it remains difficult to generate sufficient moments to protract molars after extraction of adjacent teeth due to the inherently low rigidity of ISWs.

Recently, a new method for mandibular first molar protraction was proposed [12]. An ISW in combination with long hooks was set and protract the molar from the long hook. This system may deliver forces close to the center of resistance of the teeth, reducing uncontrolled tipping. When the mandibular second premolar is extracted, the mandibular first molar must be carefully protracted to prevent mesial tipping. Bodily movement of the mandibular molars poses additional challenges compared to maxillary molars due to the unique anatomical characteristics of the mandible. It comprises dense cortical bone coupled with coarse trabecular bone, and mandibular molar roots are wide in the buccolingual direction [13]. Mesial tipping of the mandibular first molar frequently results in an undesirable vertical control, which causes downward and backward rotation of the mandible and compromised treatment results [14]. Therefore, it is important to achieve bodily movement of the mandibular molars during treatment.

Various biomechanical methods, such as shoehorn loops and cherry loops, have been employed to achieve mandibular molar bodily movement [15]. Closing loop mechanics are associated with small activations and rapid force decay, including intermittent force delivery [16]. Studies on force constancy suggest a light, continuous force may be more biologically compatible than an intermittent heavy force [17,18]. The simplicity of the ISW-based approach eliminates the need for conventional loop designs, enabling well-controlled mesialization of the molars [19]. The mechanics of ISW with long hooks in mandibular molar uprighting with mesial movement allows clinicians to achieve efficient tooth movement with minimal archwire manipulation. The objective of this study was to measure and compare the force and moment generated during mandibular molar protraction using ISW combined with short versus long hooks under simulated crowded dentition conditions. Although it is well established that space closure is commonly performed using rectangular wires, this study focused on biomechanical differences in force systems related to the novel use of ISW with varied hook lengths, aiming to elucidate their effect on controlled molar movement.

## Materials and methods

Experiment 1: measurement of force and moment applied to three teeth without crowding

**Figure 1:**
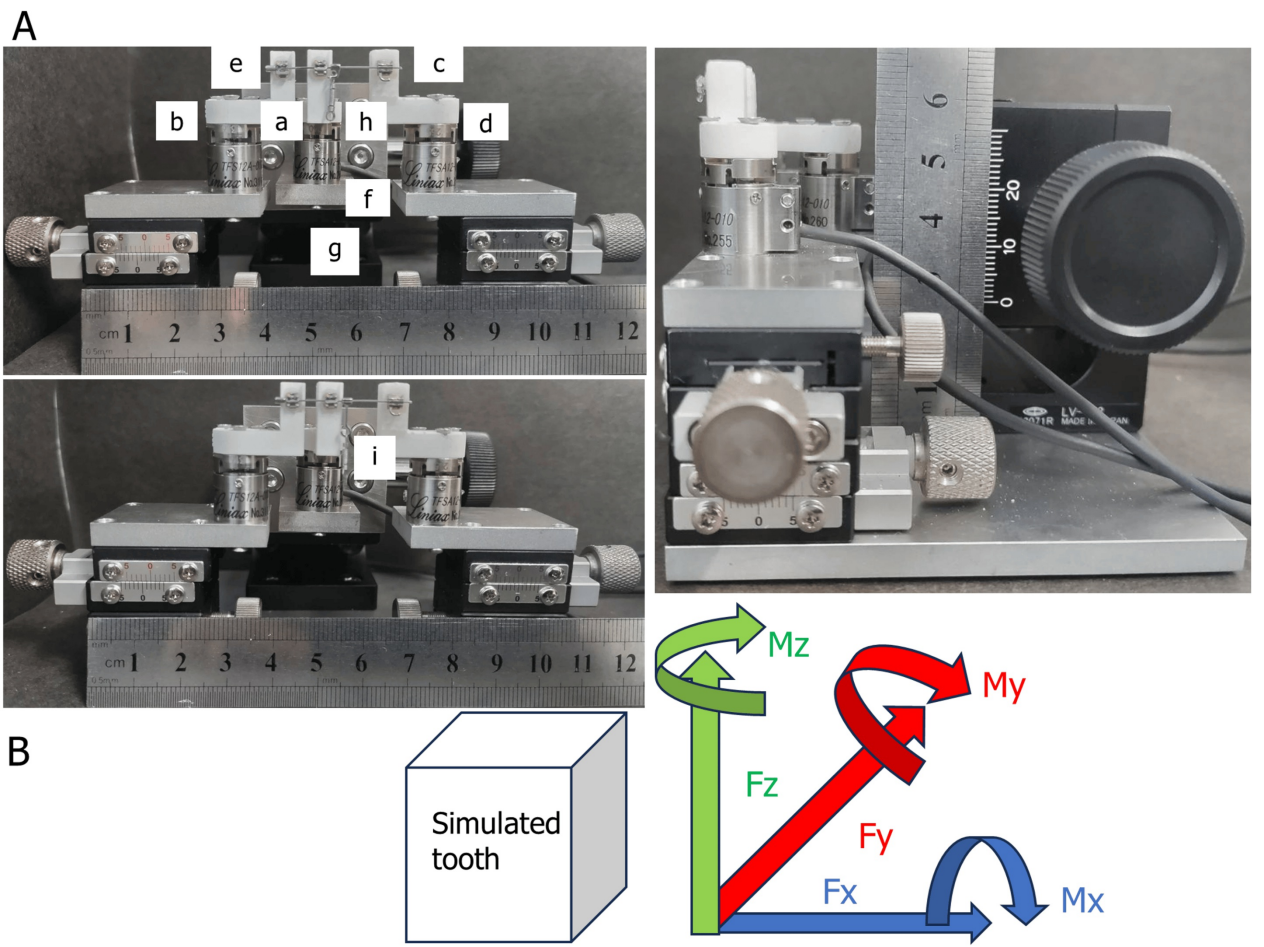
Experimental setup and coordinate definitions. (A) Photographs of the orthodontic force measurement system with three simulated teeth (canine, first premolar, and first molar) rigidly fixed to the base. The resin teeth were fabricated with a 3D printer based on CAD data and mounted without simulated periodontal ligament properties. The setup includes the following: a, self-ligating brackets with 0.018 × 0.025-inch slots bonded to the canine and first premolar, and a tube bonded to the first molar; b, 3D-printed simulated teeth; c, a 0.016 × 0.022-inch rectangular Ni-Ti wire; d, six-axis force sensors; e, crimpable stops; f, a 150-gf Ni-Ti closed coil spring; g, a vertically adjustable metal platform (1.0 mm scale) for positioning and simulating vertical displacement (e.g., crowding); h, a short hook; and i, a long hook (manually bent stainless steel wire, details in text). (B) Force and moment directions: x-axis (horizontal force to the right), y-axis (force from back to front), z-axis (upward force), and corresponding moment directions.

A tooth model simulating the mandibular canine, first premolar, and first molar was designed using CAD software and fabricated using a 3D printer with rigid resin material. This three-teeth model allowed for localized measurement of force and moment without the complexity of full-arch interactions. The teeth were fixed rigidly to the base without periodontal ligament (PDL) simulation or mobility to ensure controlled and reproducible measurement conditions. Self-ligating brackets (Mini Clippy; 0.018 × 0.025-inch slot for lower incisors, angulation 0°, torque 0°; Tomy International Inc.) were bonded to the simulated canine and first premolar, and a molar tube (0.018 × 0.025-inch slot for the first mandibular molar, angulation 2°, torque 1°; Tomy International Inc.) was attached to the simulated first molar using a self-curing dental adhesive resin cement (Orthomite Super-Bond, Sun Medical, Shiga, Japan). Self-ligating brackets were usually used to avoid biases during ligation. The three bracket slots were oriented in a straight line, parallel in all three planes, using an SS wire as a guide during the bonding procedure. To minimize bias, the bracket bonding and hook placement were standardized using a consistent protocol and performed by a single calibrated operator. Although the procedure was not blinded, care was taken to avoid placement variability using visual guides and positioning jigs. The distance between the canine and first premolar brackets was 7.0 mm, and the distance between the first premolar and first molar brackets was 16.5 mm, simulating the intraoral bicuspid extraction space [20]. The brackets were specifically designed for mandibular bicuspids, ensuring that no force or moment was generated when a straight wire was placed. A 0.016 × 0.022-inch ISW was then ligated into the brackets. Two prefabricated hooks were used and attached 2.0 mm distal to the first premolar bracket: a crimpable hook (i.e., short hook; 2 mm in height) and a long hook (8 mm in length), both manufactured by OrthoDentram (Tokyo, Japan). Both hooks were made of stainless steel. All hooks were positioned vertically and carefully aligned by an experienced orthodontist to ensure consistent angulation across trials. And a 150-gf Ni-Ti closed coil spring was connected between the hook and the molar tube to retract them (Figure 1A). A single force level of 150 gf was selected based on the typical force required for molar retraction in order to minimize confounding factors and ensure repeatability. When the Ni-Ti closed coil spring was applied, a moment was generated at the center of resistance. The spring was positioned at the shortest possible distance between the first premolar and first molar to maintain consistent force application and ensure repeatability. The moment generated at each bracket was measured five times, and the average value was calculated. The total root length was defined as the distance from the lower end of the simulated tooth to the sensor's center. Considering that the center of resistance of the tooth is located one-third of the root length from the root apex [21], the position corresponding to one-third of the root apex of the simulated tooth was also set within the sensor. Measurements were conducted using a high-precision orthodontic simulator (OSIM) incorporating multiple six-axis force sensors (TFS12A-10, Nippon Liniax Co., Ltd., Osaka, Japan; size: φ12 × 15.8 × 15 h; rated capacities: Fx, Fy: 10 N; Fz: 20 N; Mx, My, Mz: 150 N·mm; linearity error <1%) in the same way as in previous studies [11,22-24]. The measurements were acquired under static conditions, immediately following force application, without simulating time-dependent biological responses such as tooth movement or remodeling. Although the data were recorded in real time at a sampling rate of 100 Hz throughout the loading procedure, the analysis focused on the initial force and moment values captured upon application. Sensor calibration was performed prior to each experimental run. The x-axis is a positive horizontal force to the right, the y-axis is a positive force perpendicular to the x-axis from the back to the front, the z-axis is a positive force perpendicular to the xy-plane and upward, and the moment had a positive rightward direction in relation to the axis (Figure 1B). The measurement system was calibrated according to the manufacturer's instructions before each experiment to ensure accuracy, and the linearity error of the sensors was confirmed to be less than 1%, as reported in prior studies [11,22-24]. All measurements were performed under a controlled temperature environment. A transparent acrylic chamber with digital thermostatic control maintained the internal temperature at 37.0°C ± 0.2°C. The Ni-Ti wire and all components were preconditioned to this temperature before loading to simulate intraoral conditions and eliminate temperature-induced variability in wire behavior.

Experiment 2: measurement of force and moment applied to three teeth with crowded dentition

**Figure 2:**
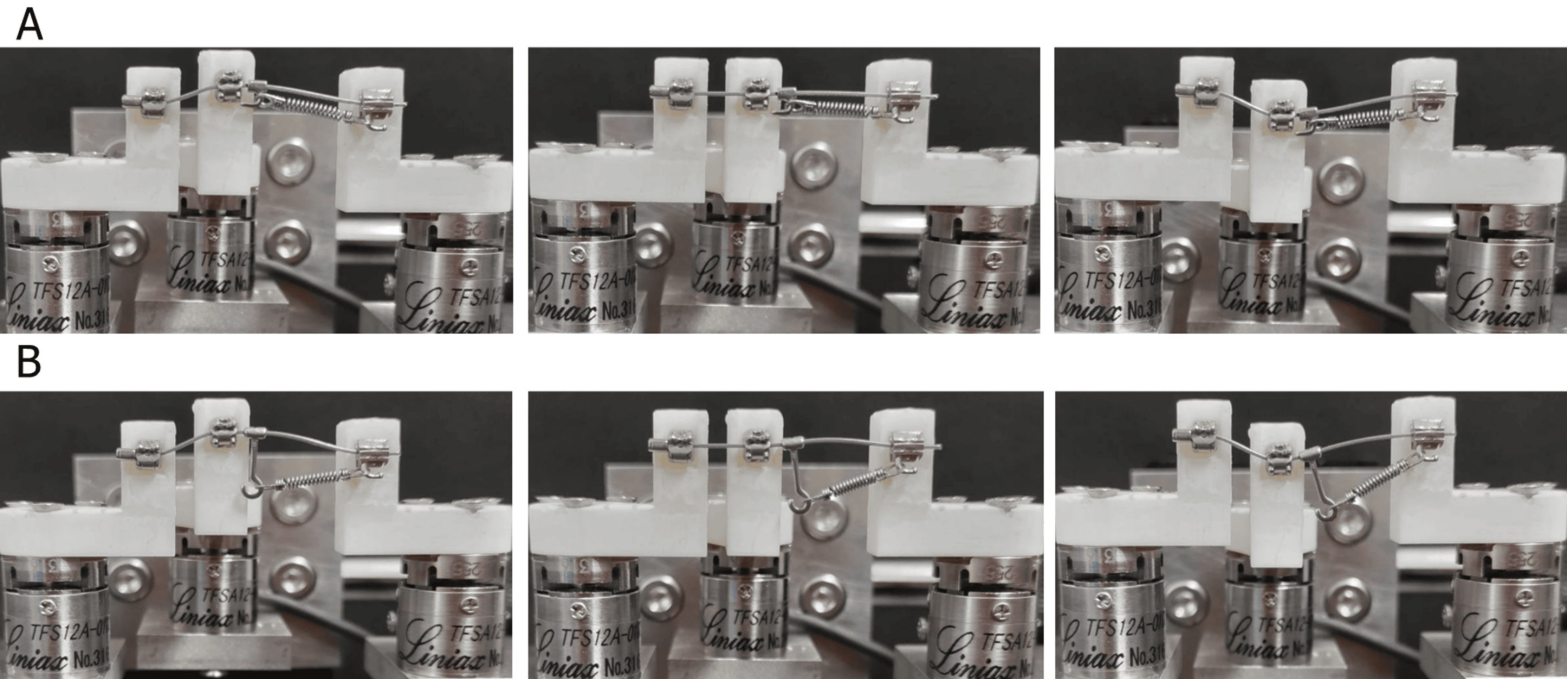
Orthodontic simulator with two types of hooks used for force application. A wire was placed next to the simulated premolar, and a 150-gf closed coil spring was placed between the hook and the tube. (A) Short hook. (B) Long hook.

The teeth were mounted on vertically adjustable platforms with a 0.1-mm increment scale, enabling precise vertical positioning. To simulate crowded dentition after mandibular second premolar extraction, the first premolar was moved 2.0 mm upward in the model. A 150-gf Ni-Ti closed coil spring was placed between the short hook and the first molar. The simulated first premolar was vertically repositioned in 0.5-mm increments both above and below its original position, up to 2.0 mm in each direction (Figure 2A). To compare the differences between the long hook and the short hook, a second set of experiments was conducted, replacing the short hook with the long hook (Figure 2B). As in experiment 1, all bracket and hook placements were conducted by the same calibrated operator following a predefined standardized protocol using mechanical positioning guides to reduce placement variability. Although the procedures were not blinded, care was taken to ensure consistency in model preparation and measurement settings. The measurement system was recalibrated before each trial to maintain accuracy.

Experiment 3: measurement of force and moment applied to three teeth depending on the position of the long hook

**Figure 3:**
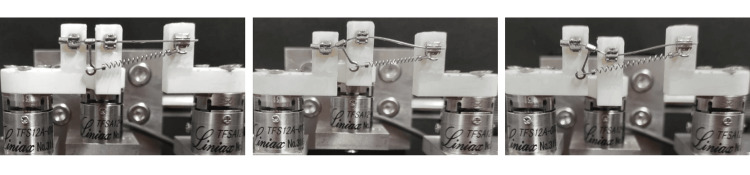
Orthodontic simulator with alternative placement of the long hook. A 150-gf closed coil spring was applied between the hook and the tube. The long hook was placed between the canine and the premolar.

To investigate the effect of hook placement, the long hook was attached 2.0 mm distal to the canine bracket. Subsequently, the first premolar was moved 2.0 mm upward and backward to simulate a different alignment scenario (Figure 3). As in experiments 1 and 2, all experimental setups, including bracket bonding and hook positioning, were performed by a trained operator using standardized guides to ensure reproducibility. While blinding was not implemented, procedural consistency was maintained. The six-axis force sensor was calibrated before measurements to verify accuracy across repeated trials.

Statistical analysis

A well-trained orthodontist inserted the wire into the brackets, and the measurements were taken five times each. In experiments 1 and 3, a paired t-test was applied to compare the forces and moments generated in the simulated teeth. In Experiment 2, linear regression analysis was conducted to determine the correlation between the height of the brackets and the magnitude of the moment and the force. A p-value of less than 0.05 was considered statistically significant. All statistical analyses were performed using Excel (Microsoft Excel for Office 365, Microsoft Corp., Redmond, WA, USA). To confirm the reproducibility of the measurement system, each measurement was repeated five times, and the standard deviations were confirmed to be within acceptable limits across all trials.

## Results

Experiment 1

**Figure 4:**
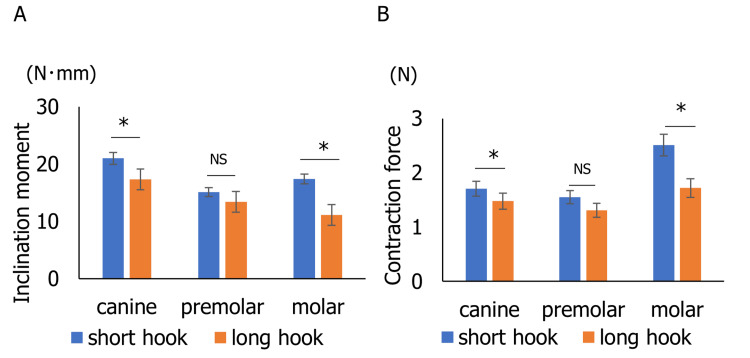
Inclination moment (My) and contraction force (Fx) exerted on the simulated canine, first premolar, and first molar. A) Inclination moment (My). (B) Contraction force (Fx). NS, not significant. *p<0.05.

Inclination moment (My) (N･mm) and contraction force (Fx) (N) generated in the canine, first premolar, and first molar were measured five times each, respectively (Figure 4). When a short hook was used, My was 21.0 ± 1.65 N·mm for the canine, 15.1 ± 1.71 N·mm for the first premolar, and 17.4 ± 1.67 N·mm for the first molar (Figure 4A). In contrast, the long hook yielded lower values: 17.3 ± 2.51 N·mm for the canine, 13.4 ± 2.32 N·mm for first premolar, and 11.1 ± 2.40 N·mm for the first molar. The My for the canine (t(7.75) = 2.63, p = 0.032) and first molar (t(7.97) = 4.99, p = 0.0011) was significantly larger with the short hook than the long hook. Furthermore, using a short hook, Fx was 1.71 ± 0.22 N for the canine, 1.55 ± 0.19 N for the first premolar, and 2.51 ± 0.23 N for the first molar (Figure 4B). On the other hand, using a long hook, Fx was 1.48 ± 0.42 N for the canine, 1.31 ± 0.38 N for the first premolar, and 1.72 ± 0.39 N for the first molar. The Fx for the canine (t(6.80) = 2.50, p = 0.042) and first molar (t(6.62) = 4.34, p = 0.0041) was significantly larger with the short hook than the long hook.

Experiment 2

**Figure 5:**
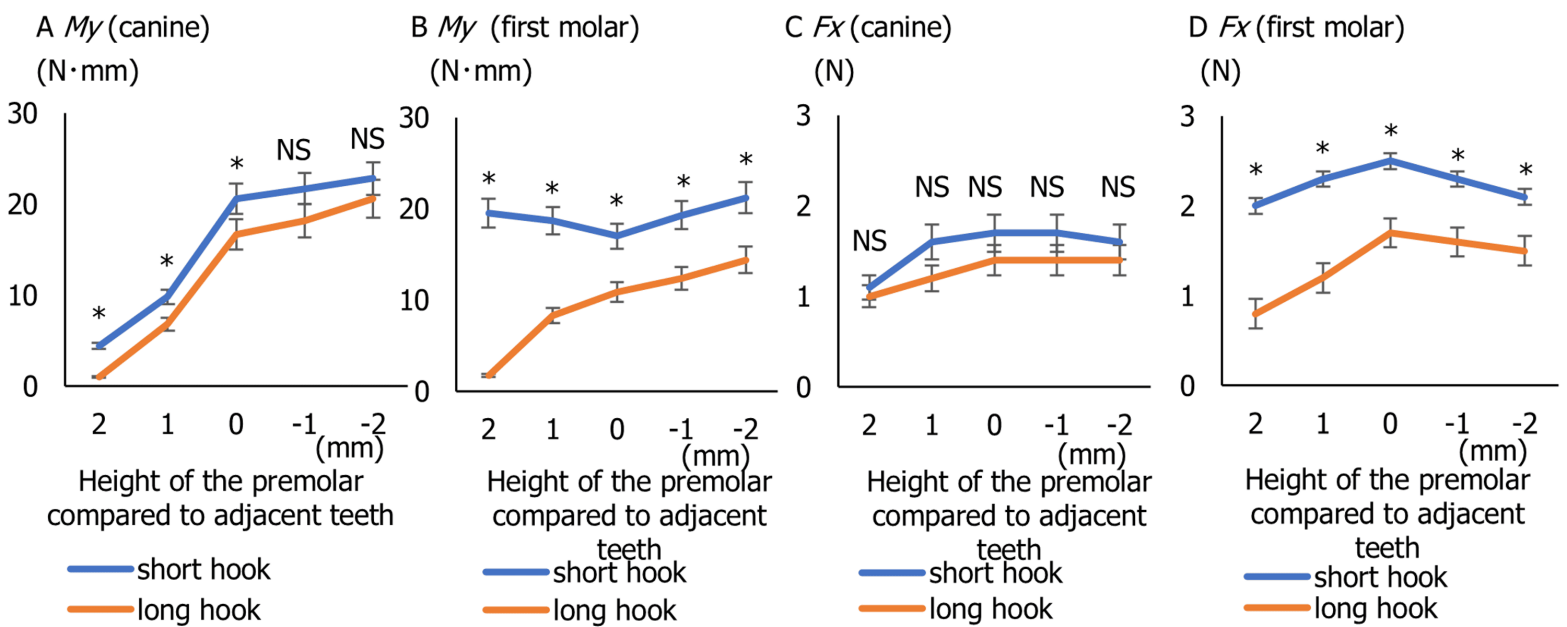
Inclination moment and contraction force affected by vertical displacement of the first premolar. Vertical axis represents the inclination moment (My) and contraction force (Fx) under different displacements of the first premolar. Horizontal axis represents the movement of the first premolar from 2.0 mm higher to 2.0 mm lower than the canine and first molar. (A) Inclination moment (My) at the canine. (B) Inclination moment (My) at the first molar. (C) Contraction force (Fx) at the canine. (D) Contraction force (Fx) at the first molar. *Indicates a statistically significant difference (p < 0.05) NS, not significant

Figure 5A shows the relationship between the height of the premolar compared to the adjacent teeth and My of the canine. When pulling the molar using a short hook, My of the canine increases as the position of the first premolar moves from the low to the high position. Similarly, when using a long hook, My increases as the first premolar moves to the high position. Using the long hook, the My of the canine was significantly smaller compared to the use of short hook when the premolar was higher by 2 mm and 1 mm than the adjacent teeth or even to the adjacent teeth (t(7.8) = 5.53, p = 0.0006), but there was no significant difference when the premolar was lower by 1 mm and 2 mm than the adjacent teeth. Figure 5B shows the relationship between the height of the premolar compared to the adjacent teeth and My of the first molar. There was a trend that My of the first molar was the least when the first premolar is at the same height as the canine and first molar. Using the long hook, the My of the first molar was significantly smaller compared to the use of short hook irrespective of the height difference with the adjacent teeth (t(8.1) = 3.92, p = 0.0045). Finally, Figures 5C, 5D show the Fx applied to the canine and first molar. In all the situations presented, Fx was 0.7-2.0 N for the canine and 0.7-2.6 N for the first molar. Although there was no difference in Fx applied to the canine with the short and long hooks (t(6.9) = 0.92, p = 0.39), the use of the long hook significantly reduced the Fx applied to both the first molar compared to the short hook (t(7.5) = 3.45, p = 0.009).

Experiment 3

**Figure 6:**
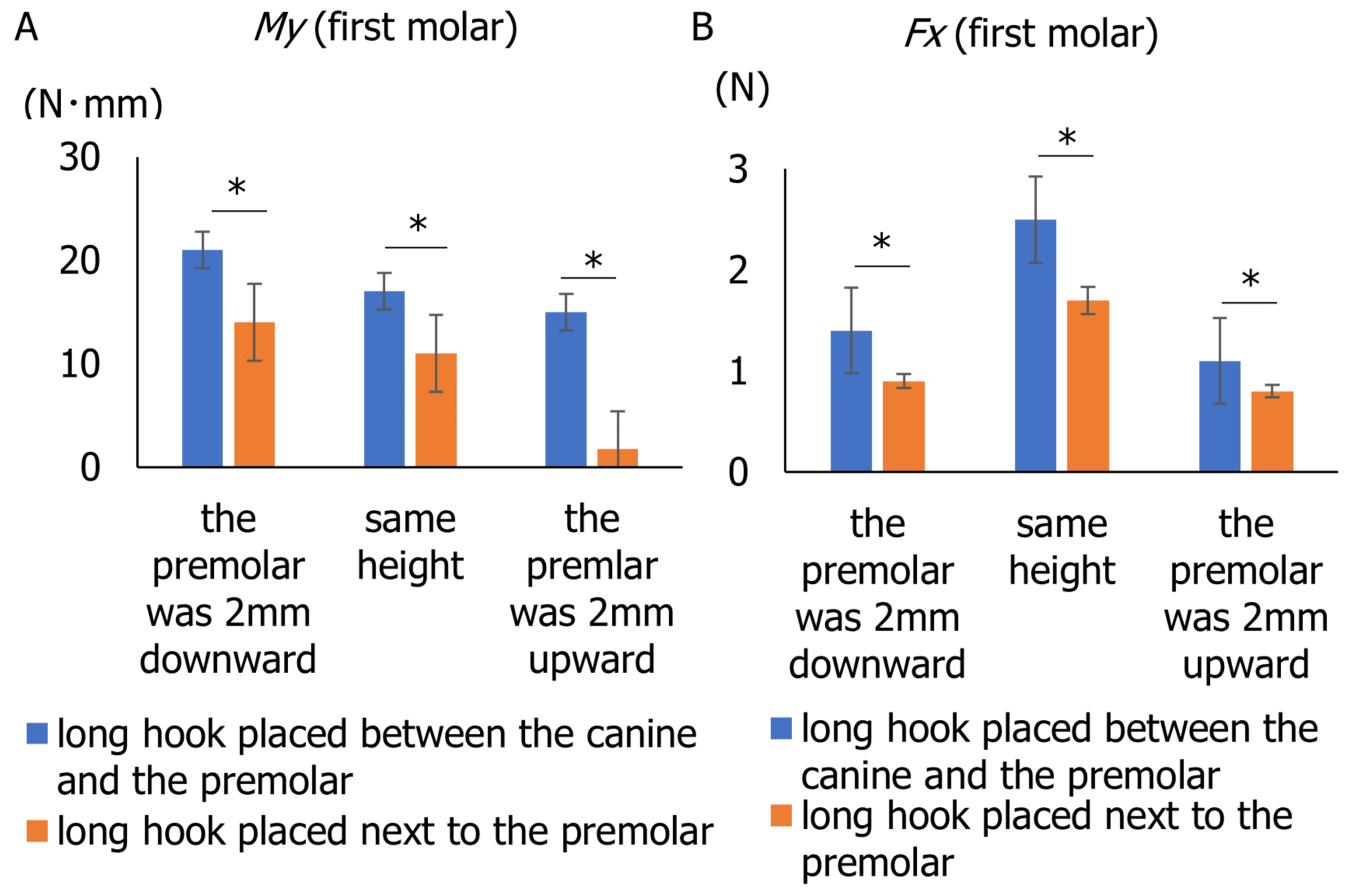
Inclination moment (My) and contraction force (Fx) at the first molar under two different hook placements (mesial and distal to the premolar) and three vertical premolar positions. The vertical axis represents the My of the first molar, and the horizontal axis shows three conditions. (A) Inclination moment (My). (B) Contraction force (Fx).

In both experiments 1 and 2, a long hook was placed on the distal side of the first premolar. Figure 6A illustrates My applied to the first molar when the long hook is placed in two different locations with three different heights. My was significantly smaller when the long hook was placed next to the premolar than it was placed between the canine and premolar irrespective of the height difference with the adjacent teeth (2 mm lower: t(8) = 5.53, p = 0.0003; same height: t(8) = 4.95, p = 0.0006; 2 mm higher: t(8) = 9.63, p = 0.00008; Figure 6A, Table 1). Likewise, Fx was significantly smaller when the long hook was placed next to the premolar than it was placed between the canine and premolar irrespective of the height difference with the adjacent teeth (2 mm lower: t(8) = 3.91, p = 0.0022; same height: t(8) = 1.88, p = 0.0483; 2 mm higher: t(8) = 2.06, p = 0.0364; Figure 6B, Table 2).

**Table 1:** Inclination moments (My) measured at the simulated first molar under the two hook placements: mesial (between the canine and first premolar) and distal (on the distal side of the first premolar) Moment values were recorded across three types of vertical positions of the first premolar: 2 mm lower, same height, and 2 mm higher relative to adjacent teeth.

	2 mm lower	Same height	2 mm higher
Long hook placed between the canine and premolar (N·mm)	21.5±2.24	17.4±1.52	15.0±2.97
Long hook placed next to the molar (N·mm)	14.2±1.92	11.1±2.41	1.76±0.80

**Table 2:** Contraction forces (Fx) measured at the simulated first molar under the same conditions described in Table 1 Hook placements included mesial and distal positions relative to the first premolar, with measurements obtained for three types of vertical positions of the premolar.

	2 mm lower	Same height	2 mm higher
Long hook placed between the canine and premolar (N)	1.42 ± 0.15	2.52 ± 0.74	1.10 ± 0.29
Long hook placed next to the molar (N)	0.94 ± 0.23	1.72 ± 0.58	0.82 ± 0.08

## Discussion

In a previous study using the low-rigidity ISW, it was demonstrated that applying a V-shaped bend to the wire could generate a moment that prevents the adjacent teeth from tipping, while effectively closing the extraction space [11]. In this study, it became possible to demonstrate that ISW can be used for extraction space closure even in a clinical situation by assuming three simulated teeth in a crowded dentition. Rigid wires, such as SS, cannot be set into the crowded dentition, and low rigidity wires, such as Ni-Ti, are not suitable for closing spaces. However, immediately after the premolar extraction, the regional acceleratory phenomenon occurs, which accelerates bone remodeling, making it crucial to begin closing the extraction space immediately to capitalize on this phenomenon for optimal and efficient tooth movement [25]. The extraction of the premolar initiates a localized inflammatory response that promotes bone remodeling, making tooth movement effective, especially immediately after extraction [26]. A case report on mandibular molar mesial movement using the ISW and long hook showed a successful outcome [12]; however, the mechanics had not been analyzed. In this study, we focused on the mechanical analysis of the force system involved. Following the method of a previous study [11], forces and moments exerted by the ISW on the simulated teeth were measured using OSIM. Particular attention was given to the second-order moment (i.e., My) and the horizontal contraction force (i.e., Fx), as these components are critical for evaluating mesiodistal control. Our findings suggest that space closure can be started in the early stages of treatment using the ISW combined with a long hook, generating an appropriate counter moment to the molars.

In experiment 1, when the molar was protracted using a short hook, the measured My at both the canine and first molar was higher compared to the condition using a long hook (Figure 4A). These findings suggest that using a short hook increases the tendency of molar tipping compared to using a long hook. These results suggest that combining the ISW with a long hook can effectively reduce the second-order inclination moment, thereby contributing to a more controlled, bodily tooth movement during molar protraction.

In experiment 2, My was measured in both the canine and the molar under the conditions simulating vertical displacement of the first premolar. When the premolar was positioned 2 mm lower than the adjacent teeth, My increased in the canine, indicating a greater tipping tendency. This movement was observed in both the short hook and long hook configurations; however, My values were consistently lower when the long hook was used (Figure 5A, Table 1). These results suggest that the use of a long hook reduces the inclination moment even in crowded dentition, especially when the premolar is in a lower position. As lower premolar positioning increases the risk of canine tipping, the long hook may be particularly effective in stabilizing tooth movement under such conditions.

In experiment 3, My was measured at the first molar, with the long hook placed either mesial or distal to the first premolar. Across all premolar height conditions, My was consistently lower when the hook was positioned distal to the premolar (Figure 6A). These findings indicate that the long hook should be placed distal to the premolar rather than mesial to the premolar. This placement allowed for a more efficient counter moment on the molar, preventing collapse into the extraction space during the movement (Figure 6A).

Considering the direction of contraction when the molar is protracted from the short hook, the force is applied horizontally to the wire. On the other hand, when the molar is protracted from the long hook, the traction point of that is positioned 8 mm below the wire, causing the force components to act both horizontally and downward. Consequently, a reduction in the traction force was expected when compared to the short hook; however, the results showed no significant difference when the short hook was replaced with the long hook. Our findings indicated that replacing the short hook with a long hook allowed effective contraction forces to be applied to the mandibular molars. On the other axis such as Mx, the moment values appeared to be influenced by the torque already applied to the bracket, resulting in relatively small values, even when the contraction force was applied. Forces and moments in the Fy, Fz, and Mz directions were consistently minimal under all conditions. This was due to the slight deflection of the wire used in the experiment. Especially, a small value of the Fz seemed to be beneficial because occluso-gingival force causes the extrusion or intrusion of the mandibular molars. These vertical forces can disrupt the vertical relationship, and the extrusion of the canines can lead to bite deepening.

In clinical practice, mandibular molar protraction can be achieved by either using a Ni-Ti closed coil spring or lacing with a ligature wire between the canine and the first molar. The latter, known as “lace back technique”, is often considered a more feasible alternative due to its simplicity and adjustability [27]. However, in this experiment, a 150-gf Ni-Ti closed coil spring was used to activate the system, as it provides a more controlled force. A previous study demonstrated that contraction forces of 100 gf or 150 gf were most effective with Ni-Ti wire, as greater forces did not increase the generated moment and could cause unwanted effects such as molar tipping or buccal displacement. A 50 gf was too low to retract teeth, and 200 gf may generate too many side effects such as crown tipping or buccal displacement of the molar [23]. A coil spring is particularly suitable for anterior retraction because it allows the wire to move uniformly backward. In contrast, lace back is also used for space closure. This is because a laced ligature maintains the deflection of the archwire as the teeth move along the wire, continuously applying a moment to the teeth. Furthermore, controlling the amount of activation is crucial, as clinicians can reduce activation when the target teeth become excessively tipped during space closure, similar to adjustments made with loop mechanics. Therefore, careful consideration of whether to use the Ni-Ti coil spring or the laced ligature wire is important depending on the clinical situation.

AAlthough OSIM has only been tested in vitro, and the experimental conditions do not fully replicate clinical situations-particularly due to absence of periodontal ligament and alveolar bone-it still provides valuable insights into orthodontic force systems. In particular, the lack of simulation of the PDL and alveolar bone in this in vitro setup limits the direct translatability of the results to clinical scenarios. The actual biomechanical behavior in vivo may differ due to the presence of these structures, which contribute significantly to tooth movement dynamics and stress distribution. Therefore, the outcomes should be interpreted as preliminary mechanical findings rather than direct clinical recommendations. This study primarily focused on efficient treatments for crowded dentition with an upward or downward premolar. In clinical practice, buccal tilting of the crown is commonly observed during retraction of teeth into a linguo-version to achieve an ideal arch form. To manage this issue, various treatment techniques, such as applying third-order bends, are recommended for generating lingual torque on the crown. Therefore, our next step will involve assessing and developing treatment options for other types of crowding, expanding the application of this approach. Future studies are needed to explore the effects of different combinations of wire sizes and slot sizes on the orthodontic forces and moments applied to the teeth. Although the results demonstrated statistically and mechanically significant differences among various configurations, the findings are limited by the in vitro nature of the study and cannot be directly extrapolated to clinical outcomes without further in vivo validation. Future studies incorporating animal models or clinical trials are essential to evaluate the biological responses and practical efficacy of this orthodontic approach. Additionally, to enhance the clinical relevance of future experiments, the anatomical arrangement of teeth must be considered, for instance, the second molar is located posterior to the first molar, and the anterior teeth are positioned anterior to the canine. Experiments will be designed to more closely simulate these clinical conditions. Moreover, since the root surface area and center of resistance vary according to tooth type, a new experimental system will be developed to integrate these factors and determine the optimal orthodontic force and moment for each tooth type.

## Conclusions

Our measurement-based analysis suggested that using an ISW with a long hook generated a sufficient moment to control molar inclination, preventing tipping and allowing effective mesial protraction. Although these findings were derived from an in vitro model that lacks the PDL and alveolar bone, they provide important insights into how clinicians might achieve space closure without resorting to the rigid SS wire. This method has the potential to simplify treatment mechanics by allowing continuous use of a single archwire system from alignment to space closure, potentially reducing chair time and patient discomfort. Further in vivo validation is warranted, but the approach may serve as a useful alternative in clinical settings, especially for cases involving early stage protraction of mandibular molars in crowded dentitions.
